# Addition of Nimotuzumab to Standard TPF Regimen in Locally Advanced Head and Neck Cancer: A Single Institutional Study

**DOI:** 10.1155/2021/6641963

**Published:** 2021-04-16

**Authors:** Samuel Luke Koramati, Vinu Sarathy, Hrishi Varayathu, Beulah Elsa Thomas, Radheshyam Naik

**Affiliations:** ^1^Department of Clinical Pharmacology, Healthcare Global Enterprises Limited, Bengaluru, Karnataka, India; ^2^Department of Medical Oncology, Healthcare Global Enterprises Limited, Bengaluru, Karnataka, India; ^3^Department of Translational Medicine and Therapeutics, Healthcare Global Enterprises Limited, Bengaluru, Karnataka, India

## Abstract

**Background:**

Induction docetaxel, cisplatin, and 5-fluorouracil (TPF) chemotherapy followed by definitive concurrent chemoradiation remains the standard of care in locally advanced squamous cell carcinoma of head and neck cancers despite which the survival remains low. So, we analyzed the efficacy and adverse effect profile of the addition of nimotuzumab to standard TPF induction chemotherapy. Methods. We included 20 patients with locally advanced squamous cell carcinoma of the head and neck. Patients were administered with induction chemotherapy with nimotuzumab plus docetaxel, cisplatin, and 5-fluorouracil (TPF + N) followed by definitive concurrent chemoradiation with carboplatin. Treatment responses were assessed by PET-CT following induction chemotherapy and concurrent chemoradiation. Response rates, survival, and adverse effects data were tabulated and analyzed using the Kaplan Meier method.

**Results:**

At a minimum follow-up of two years, the median progression-free survival (PFS) and median overall survival (OS) were 16 months and 38 months, respectively. PFS and OS were not reached (NR) in patients who showed a complete radiological response (CR). Median PFS and OS in patients who had partial response were 17.6 and 34.5 months, respectively. All subsites of primary including oral cavity, hypopharynx, and oropharynx showed similar response rates and survival. Overall the treatment was well tolerated with predominantly grade 1/2 toxicities.

**Conclusions:**

Patients with locally advanced head and neck cancer could possibly have a better response and survival with nimotuzumab added to the standard TPF regimen. A complete response may serve as a good surrogate for survival irrespective of the primary site of head and neck cancer.

## 1. Introduction

Head and neck cancer, especially oral cancer, ranks second in incidence and mortality in India [1]. Cigarette smoking and alcohol are the established causes of head and neck cancer in the Western population [2] while smokeless tobacco, betel nut chewing, and Epstein–Barr virus are the chief etiologies in India [3–5]. Additional factors like poor education/literacy and socioeconomic status also play a major contribution towards the increased incidence of advanced head and neck squamous cell cancer (HNSCC) in India [6]. 60% of patients diagnosed with HNSCC in India present with locally advanced disease (stage III in 39% and stage IV in 23%) further contributing to increased mortality. References [6, 7].

Concurrent definitive chemoradiation is the standard treatment option for locally advanced HNSCC. In recent times, docetaxel, cisplatin, and 5-fluorouracil (TPF) have been increasingly used as an effective induction regimen based on the survival benefit obtained from the landmark TAX-323 and TAX 324 trial. However, its role in inoperable disease is a debatable topic over standard concurrent definitive chemoradiation (CRT) especially in oral cancers [8–10].

More than 90% of HNSCC overexpress epidermal growth factor receptor (EGFR) which is also a poor prognostic marker [11]. Cetuximab, an anti-EGFR monoclonal antibody has been approved for upfront treatment of locally advanced HNSCC concurrently with radiation and also in the recurrent metastatic setting along with cisplatin and 5-FU^.^ However, the addition of cetuximab was associated with significantly increased toxicities such as dermatitis, hypomagnesemia, skin rash, and sepsis [12, 13]. Nimotuzumab is yet another anti-EGFR agent which has shown tremendous benefit with minimal added toxicity when used along with CRT in locally advanced HNSCC [14]. Nimotuzumab has also shown greater response rates with no added toxicity when used as an induction agent long with platinum and 5-FU in locally advanced nasopharyngeal cancer [15].

Despite the various sequencing and combination treatment strategies, the median survival for locally advanced HNSCC has remained dismal with an average of 19 months [9]. Since nimotuzumab showed a favorable safety and efficacy profile in the above studies, our study assessed the benefit and adverse effect profile of adding nimotuzumab to standard induction TPF regimen in locally advanced HNSCC. Based on a search using PubMed and Embase and to our knowledge, this is the first study analyzing the addition of nimotuzumab to induction TPF in locally advanced HNSCC to date.

## 2. Materials and Methods

The study was conducted in HealthCare Global Hospital, Bangalore, after approval from HCG-Central Ethics Committee (Reg. no. ECR/386/Inst/KA/2013/RR-19). 20 locally advanced HNSCC patients aged >18 years from August 2012 to July 2017 were enrolled and followed up till March 2020. Treatment naive patients with unresectable locally advanced disease and tumor–node–metastasis (TNM) stage of III or IV without metastases with a performance status (PS) of 1 or less were included in our study. Patients with tumors of the nasopharynx, nasal, and paranasal cavities were excluded.

### 2.1. Treatment Protocol

The following was the treatment protocol used for patients.

Nimotuzumab was given at a dose of 200 mg intravenously on day 1. Docetaxel (75 mg/m^2^) day 1, cisplatin (75 mg/m^2^) day 1, and 5-FU (750 mg/m^2^) days 1–5 were administered intravenously every 21 days for 3 cycles. After 3 cycles of induction TPF + nimotuzumab (TPF + N), the response was assessed with PET-CT and all patients proceeded to concurrent chemoradiation (CRT) with carboplatin. Another PET-CT was done 8 weeks after CRT to evaluate the response. Those patients with the residual disease were given the option to undergo salvage surgery.

### 2.2. Evaluation of Treatment

Radiological response assessment was done by RECIST (version 1.1) based on PET-CT SCAN imaging modality. Adverse effects were graded based on NCI- CTCAE version 4.0 [16].

### 2.3. Statistical Analysis

SPSS version 23 was used for data analysis. Frequencies and percentages are reported for categorical variables and the continuous variables were expressed as mean and standard deviation for normally distributed data and median and range for skewed data. Kaplan Meier survival analysis was carried for progression-free survival. Response rates were evaluated by using Chi-Square or Fischer's test. Results are graphically represented where deemed necessary. Probability values below 0.05 were considered statistically significant.

## 3. Results

### 3.1. Patient Characteristics

A total of 20 patients were included which comprised 11 males and 9 females. The median age was 54 years (range: 18–75 years). Most patients had a good ECOG (eastern cooperative oncology group) performance of 1 (95%). Most of our study patients had no comorbidities (65%) while type 2 diabetes and hypertension were present in the rest (35%). Half the patients had no ill habits while tobacco and alcohol consumption constituted the rest (50%). Oral cancer patients were the majority comprising 55% while hypopharynx (35%) and oropharynx (10%) made up the rest. Stage III group had 40% of patients and stage IV the remaining 60%. The complete baseline characteristics are enlisted in Table 1.

### 3.2. Patients' Response Analysis

All 20 patients tolerated and completed 3 cycles of induction TPF + N followed by CRT with carboplatin. Only 3 patients with the residual disease were willing and underwent salvage surgery after completion of therapy. A complete response of 10% was achieved after induction TPF + N which further increased to 30% after chemoradiotherapy. We obtained a partial response of 35% at the end of therapy. The overall response rate after induction TPF + N and CRT was 65% which was close to significance (p-0.192). However, 25% of patients progressed despite the induction TPF + N and CRT treatment. The above responses are shown in Table 2.

### 3.3. Analysis of Patient Survival

**Figure 1:**
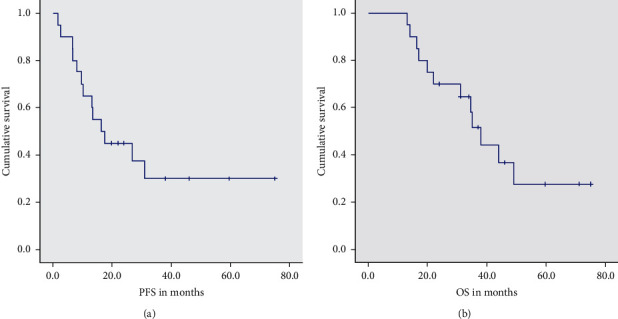
Median PFS and OS in months after induction TPF + *N* and concurrent chemoradiation.

Our study showed a median progression-free survival (PFS) of 16 months and median overall survival (OS) of 38 months. The response rates showed a positive correlation with survival data. The OS and PFS for patients with a complete response were not reached (NR). Patients who achieved a partial response had a median PFS and OS of 17.6 and 34.5 months, respectively. Patients with progressive disease after treatment had the worst PFS and OS of 6.7 months and 17 months, respectively. The complete data is depicted in Table 2 and Figure 1.

### 3.4. Survival Analysis by Anatomical Subsite

**Figure 2:**
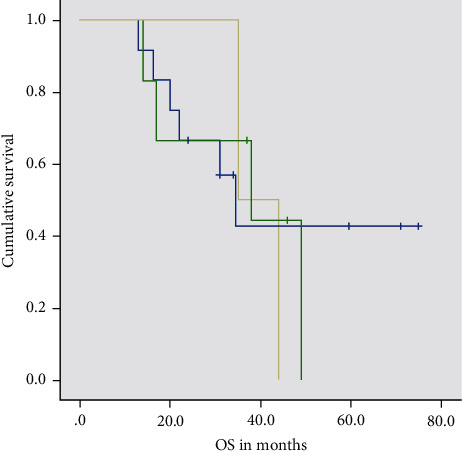
Kaplan Meier Curve of progression-free survival (months) according to subsite (blue line represents oral cavity, green line represents hypopharynx, and yellow represents oropharynx).

Median overall survival did not vary greatly with primary tumor subsite. Median OS was 34.5 months for oral cavity, 38 months for hypopharynx, and 35 months for oropharynx which was not statistically significant (p-0.918). The data is represented in Table 2 and Figure 2.

### 3.5. Adverse Effect Profile

All our patients tolerated and completed the induction and concurrent CRT as per schedule. Eight patients required dose modification due to grade 3/4 toxicity. Most toxicities were of grade 1/2 which did not require any dose modification. Most grade 3/4 toxicities occurred with nausea/vomiting (25%) and neutropenia (40%) which were managed conservatively. Grade 3/4 mucositis was present in 35% of patients possibly also contributed by concurrent CRT. Neuropathy was chiefly of grade 1/2 (30%) and grade 3 in 10% of patients. Interestingly, skin rash was present only in 10% of patients, all of whom were grade 1. None of the patients had hypomagnesemia. The complete adverse effect profile is illustrated in Table 3.

## 4. Discussion

Head and neck cancer, especially oral cancer ranks second in incidence and mortality in India with 60% of patients diagnosed in a locally advanced stage. The landmark TAX 323 trial established the role of induction TPF regimen in locally advanced HNSCC; however, the survival rates for the above group continued to be poor [9]. As nimotuzumab has shown promising activity with minimal added toxicity when combined with chemoradiation in locally advanced HNSCC, our study was designed to establish an effective safe and tolerable regimen using TPF + N as induction treatment for locally advanced HNSCC [14].

Our study showed a 10% complete response (CR) rate after induction TPF + N which further increased to 30% after the completion of chemoradiation. Our CR rates are slightly better than the study done by Vermorken et al., which had a CR of 6.6% after induction TPF and a CR of 19.9% after concurrent chemoradiation [9].

We achieved a median progression-free survival of 16 months and overall survival of 38 months. This was considerably better than the study by Vermorken et al. where the PFS and OS were 11.0 months and 18.8 months, respectively [9]. Our survival could have been possibly better as we had a higher fraction of T2 patients (55%) and possibly due to the addition of nimotuzumab to induction TPF.

Our results are also comparable to a Spanish study by Hitt et al. where paclitaxel, cisplatin, and 5-FU were used as the induction regimen. They reported a median survival of 36 months in the unresectable locally advanced HNSCC group which is similar to our study [17].

TAX 324 trial by Lorch et al. which used an induction TPF versus platinum-5FU (PF) arm showed a median overall survival of 70.6 months versus 34.8 months in the TPF and PF arms, respectively [8]. However, the above trial included both resectable and unresectable patients and had a high percentage of HPV positive patients (>50%) which could have contributed to its better median OS.

Our study showed a positive correlation between response and survival. This was demonstrated by a much superior survival in those patients achieving a radiological complete response (OS-not reached) compared to 34.5 months, 31 months, and just 17 months in those having a partial response, stable disease, and progressive disease, respectively. This is similar to a study done by Saini SK et al., who showed that response to induction chemotherapy can be a predictive marker for response to subsequent chemoradiotherapy and survival. Their study showed a hazard ratio of 0.463 for mortality in patients achieving partial response or more when compared to others with stable or progressive disease [18].

We did not find any difference in survival among the various subsites of the primary tumor. We obtained a median overall survival of 34.5 months, 38 months, and 35 months in the oral cavity, hypopharynx, and oropharyngeal tumors, respectively.

Our study is one of the first studies analyzing the effectiveness of EGFR antibodies for oral cavity cancers. Bonner et al. demonstrated the efficacy of cetuximab when used concurrently with radiation in locally advanced HNSCC. However, they excluded patients with primary of the oral cavity [19]. Similarly, the study by Patil VM et al. only included three patients with oral cavity cancer [14].

All our patients were able to complete induction TPF + N followed by CRT with carboplatin. 8 patients required dose modification due to grade 3/4 toxicity. Most adverse effects were of grade 1/2 which did not require any dose modification. Grade 3/4 toxicities mainly presented as nausea/vomiting (25%) and neutropenia (40%) which was managed conservatively. Grade 3/4 mucositis was present in 35% of patients. Mucositis was seen more commonly in patients receiving concurrent chemoradiation with carboplatin. Our adverse effect profile was slightly different from that of TAX 323 where 75% of patients had grade 3/4 neutropenia, 6% had severe diarrhea, 11.2% grade 3/4 stomatitis, and 6.7% had significant nausea or vomiting [9]. This difference can possibly be explained due to our small sample size and the difference in ethnicity of the study population.

Skin rash and hypomagnesemia reported commonly with cetuximab were seen in only 10% of patients, all of whom were grade 1. Nimotuzumab Fab fragment has a 10-fold lower affinity when compared with the cetuximab Fab fragment [20]. Unlike cetuximab, nimotuzumab requires bivalent binding to maintain stable association with EGFR on the cell surface. Hence, nimotuzumab preferentially binds EGFR on cells that have a medium to high surface density of EGFR molecules that allow for bivalent binding and binding is more monovalent and transient in cells with a low density of EGFR [21]. Unlike cetuximab, this unique property of nimotuzumab may explain its low toxicity profile.

Synergy of nimotuzumab with chemotherapeutic drugs is likely due to the mechanism of actions of both of the drugs. Chemotherapy induces DNA damage leading to cell cycle arrest in the G1 peak to facilitate repair. Blockade of EGFR at this point suppresses the signal transduction pathways required for cell proliferation and repair causing cells to undergo apoptosis [22]. Nimotuzumab being a humanized IgG1 monoclonal antibody also showed antiproliferative actions in vitro when tested on squamous cell carcinoma cultures [23]. Antiangiogenic, antimitotic, and cytotoxic effects were also seen in vivo from its dose-dependent activity on vascular endothelial growth factor (VEGF). Antibody-dependent cell-mediated cytotoxicity (ADCC) and complement-dependent cytotoxicity (ADC) are also other mechanisms by which nimotuzumab could be synergistic with chemotherapy [24].

The limitation of our study is a small sample size and single-arm design. Assessment of HPV status was not included in our study which could have influenced treatment outcome and response rates. Quality of life was not recorded which may have helped in further analysis of effectiveness.

## 5. Conclusion

The addition of nimotuzumab to induction TPF could be a very effective option increasing response rates and survival with no added toxicity. Even the subset of patients with oral cavity primary tumor could reap the benefits of nimotuzumab added to standard induction as demonstrated in our study. Complete remission to induction and chemoradiation may well be a surrogate for overall survival. Larger randomized multicentric studies will be needed to confirm the same.

## Figures and Tables

**Table 1:** Baseline characteristics (data are expressed in median, range, and number or percentage).

Variables	*N* = 20
Median age (range)	54 (18-75)
*Gender (%)*	
Male	11 (55%)
Female	9 (45%)

*Performance status (%)*	
PS- 1	19 (95%)
PS-2	1 (5%)

*Comorbidities (%)*	
No comorbidities	13 (65%)
Hypertension	02 (10%)
Diabetes mellitus	02 (10%)
Hypertension + diabetes mellitus	03 (15%)

*Habits (%)*	
No habits	10 (50%)
Tobacco products	06 (30%)
Tobacco + alcohol	04 (20%)

*Site of cancer (%)*	
Oral cavity	12 (60%)
Hypopharynx	06 (30%)
Oropharynx	02 (10%)

*Tumour (T) and nodal (N) stage (%)*	
T2	11 (55%)
T3	03 (15%)
T4	06 (30%)

*N (%)*	
N0	01 (5%)
N1	09 (45%)
N2	10 (50%)

*Stage (%)*	
Stage III	08 (40%)
Stage IV	12 (60%)

**Table 2:** Overall analysis of study population (data are expressed in median, range, and number or percentage; PFS, progression-free survival; OS, overall survival).

Variables	*N*	Median PFS	Median OS	*P* value
*Overall*	20	16 months (7.2–20.6 months)	38 months (32–44 months)	
Anatomic site				
Oral cavity	12 (60%)		34.5 months	
Hypopharynx	6 (30%)		38 months	
Oropharynx	2 (10%)		35 months	

*Overall response rate after induction*	75%			*P* < 0.001
Complete response	2 (10%)			
Partial response	13 (65%)			
Stable disease	2 (10%)			
Progressive disease	3 (15%)			

*Overall response rate after CRT*	65%			*P* − 0.192
Complete response	6 (30%)	Not reached	Not reached	
Partial response	7 (35%)	17.6 months	34.5 months	
Stable disease	2 (10%)	8.1 months	31 months	
Progressive disease	5 (25%)	6.7 months	17 months	

**Table 3:** Adverse effect profile of induction (TPF + *N*) and concurrent chemoradiation (T: docetaxel; P, cisplatin; F-5 FU, N-nimotuzumab).

Adverse effects	*N* = 20
*Nausea/vomiting*	
Grade I	4 (20%)
Grade II	6 (30%)
Grade III	2 (10%)
Grade IV	3 (15%)

*Mucositis*	
Grade I	8 (40%)
Grade II	2 (10%)
Grade III	4 (20%)
Grade IV	3 (15%)

*Rash*	
Grade I	2 (10%)

*Fatigue*	
Grade I	7 (35%)
Grade II	4 (20%)
Grade III	1 (5%)

*Diarrhea*	
Grade 1	5 (25%)
Grade II	3 (15%)

*Neutropenia*	
Grade I	5 (25%)
Grade II	7 (35%)
Grade III	3 (15%)
Grade IV	5 (25%)

*Neuropathy*	
Grade I	4 (20%)
Grade II	2 (10%)
Grade III	2 (10%)

## Data Availability

The data are available from the corresponding author upon request.
